# FRED (A Framework for Reconstructing Epidemic Dynamics): an open-source software system for modeling infectious diseases and control strategies using census-based populations

**DOI:** 10.1186/1471-2458-13-940

**Published:** 2013-10-08

**Authors:** John J Grefenstette, Shawn T Brown, Roni Rosenfeld, Jay DePasse, Nathan TB Stone, Phillip C Cooley, William D Wheaton, Alona Fyshe, David D Galloway, Anuroop Sriram, Hasan Guclu, Thomas Abraham, Donald S Burke

**Affiliations:** 1 Graduate School of Public Health, University of Pittsburgh, Pittsburgh, Pennsylvania, USA; 2 Pittsburgh Supercomputing Center, Carnegie Mellon University, Pittsburgh, Pennsylvania, USA; 3 School of Computer Science, Carnegie Mellon University, Pittsburgh, Pennsylvania, USA; 4 RTI International, Research Triangle Park, Durham, North Carolina, USA

**Keywords:** Pandemic influenza, Simulator, Agent-based model, Synthetic population, Influenza modeling

## Abstract

**Background:**

Mathematical and computational models provide valuable tools that help public health planners to evaluate competing health interventions, especially for novel circumstances that cannot be examined through observational or controlled studies, such as pandemic influenza. The spread of diseases like influenza depends on the mixing patterns within the population, and these mixing patterns depend in part on local factors including the spatial distribution and age structure of the population, the distribution of size and composition of households, employment status and commuting patterns of adults, and the size and age structure of schools. Finally, public health planners must take into account the health behavior patterns of the population, patterns that often vary according to socioeconomic factors such as race, household income, and education levels.

**Results:**

FRED (a Framework for Reconstructing Epidemic Dynamics) is a freely available open-source agent-based modeling system based closely on models used in previously published studies of pandemic influenza. This version of FRED uses open-access census-based synthetic populations that capture the demographic and geographic heterogeneities of the population, including realistic household, school, and workplace social networks. FRED epidemic models are currently available for every state and county in the United States, and for selected international locations.

**Conclusions:**

State and county public health planners can use FRED to explore the effects of possible influenza epidemics in specific geographic regions of interest and to help evaluate the effect of interventions such as vaccination programs and school closure policies. FRED is available under a free open source license in order to contribute to the development of better modeling tools and to encourage open discussion of modeling tools being used to evaluate public health policies. We also welcome participation by other researchers in the further development of FRED.

## Background

Mathematical and computational models provide valuable planning tools for public health challenges, especially for novel circumstances that cannot be examined through observational or controlled studies, such as pandemic influenza [1-12] or hypothetical bioterrorist attacks [13,14]. The development of models ideally involves a close working relationship between the modeling team and the decision-maker using the model. Beyond the immediate outputs of a model itself, the modeling process itself can serve as a way of thinking through complex situations and clarifying assumptions [15]. While mathematical models have a long history of providing solid foundations for understanding disease dynamics [16], the tractability of analytic models may require neglecting heterogeneities in the population that may have important impacts on epidemic dynamics and on the effectiveness of possible interventions. For example, it has been suggested that attack rates for the 2009 H1N1 pandemic exhibited a high degree of spatiotemporal heterogeneities among different regions due to regional differences in socio-demographic factors [17]. In particular, the spread of infectious disease such as influenza depends on the mixing patterns within the population, and these patterns are in turn determined by numerous factors, including: population size and density [18,19], the age structure of the population [20], the size and composition of households [21], school sizes and schedules [6,10,22-24], demographic and socioeconomic risk factors [25] including access to health care facilities [9,11,26], employment patterns and policies [27], travel and commuting patterns [12,28], and local behavioral practices including vaccine acceptance [26,29] and personal hygiene [30]. With these considerations in mind, public health officials may have particular interest in planning tools that take into account the specific characteristics of the local population of the region under their responsibility and that permit them to compare expected outcomes within their jurisdiction with expected outcomes in surrounding communities, or across an entire state.

This article describes FRED (a Framework for Reconstructing Epidemic Dynamics), a freely available open-source epidemic modeling system that uses census-based synthetic populations to capture the demographic and geographic heterogeneities of the population, including realistic household, school, and workplace social networks. FRED models are currently available for every state and county in the United States, and selected international locations. State and county public health planners can use FRED to explore the effects of possible influenza epidemics in their regions and to help evaluate the likely effect of interventions such as vaccination programs and school closure policies.

FRED represents a major software redesign and open-source release of epidemic models used in previously published studies by our team to evaluate potential responses to influenza pandemics, including vaccination policies [5,7-9], school closure [6,10], the role of health care workers [11], and the effects of subway travel [12]. Building on these previous models, FRED was designed as a flexible framework for epidemic modeling, rather than a fixed model of a particular infectious disease. While originally designed to study influenza, FRED can be adapted to other infectious diseases, such as measles, by modifying configuration files characterizing the natural history of the disease. Other user-modifiable parameters include the initial immunological profile of the population, the availability and efficacy of vaccine and anti-viral drugs, and a flexible set of intervention policies regarding vaccine distribution, school closures and other non-pharmaceutical interventions. In addition, human behaviors in response to an epidemic can also be modeled in a variety of ways, from specifying simple probabilities that certain groups will get a vaccine or stay home from work or school when sick, to more sophisticated behavioral dynamics such as being influenced by concerns over a spreading epidemic.

## Implementation

Key features of FRED include:

• Realistic synthetic populations based on the US Census Bureau’s Public Use Microdata (PUMS) data and Census aggregated data. FRED is the first open-source epidemic model designed to use the latest synthetic US population developed by RTI [31].

• Highly modular, object-oriented software design to support rapid adaptation to a wide variety of infectious disease scenarios.

• Scalable and efficient simulation of large epidemics. FRED can be run on a variety of computer platforms from laptops to supercomputers, depending on the size of the population being simulated. Simulations of an influenza epidemic like the H1N1 pandemic in a population of 1 million people takes less than two minutes on a typical laptop computer.

• Multiple circulating strains can be simulated, making it suitable for the investigation of virus evolution, for example, antigenic drift or the evolution of resistant strains.

• Flexible ways to specify agent health behavior and decision rules*.* Agents in FRED may exhibit a number of health-related behaviors involving individual health decisions, such as staying home when sick, accepting a vaccine or taking an anti-viral drug. The FRED platform is designed to accommodate a range of models of health behavior and supports a variety of strategies to determine an agent’s willingness to adopt a behavior [27].

### Synthetic population

FRED explicitly represents every individual in a specific geographic region. For regions within the United States, FRED uses the 2005–2009 U.S. Synthetic Population Database (Version 2) from RTI International [31,32]. The synthetic population used an iterative fitting method [33] to generate an agent population from the US Census Bureau’s Public Use Microdata files (PUMS) and aggregated data from the 2005–2009 American Community Survey (ACS) 5-year sample. The synthetic population contains geographically located synthetic households and household residents for the United States, as well as group quarters locations and residents (for college dorms, prisons, nursing homes, and military bases), schools and assignments of students to schools, workplaces and assignments of workers to workplaces. Each household, group quarters, school and workplace is mapped to a specific geographic location, reflecting the actual spatial distribution of the area and the distance travelled by individuals to work or to school [34,35]. Each agent has associated demographic and socioeconomic information (e.g., age, sex, race, household income) and locations for their activities (e.g., household, neighborhood, and possibly school or workplace). The number of elements for each category in the synthetic population is shown in Table 1.

**Table 1:** Elements in US synthetic population used in FRED based on 2005–2009 American community survey (ACS)

**Individuals and interaction groups**	**Number in US synthetic population**
**Persons**	289,390,247
**Households**	112,595,578
**Workplaces**	10,696,738
**Schools**	129,329

**Figure 1:**
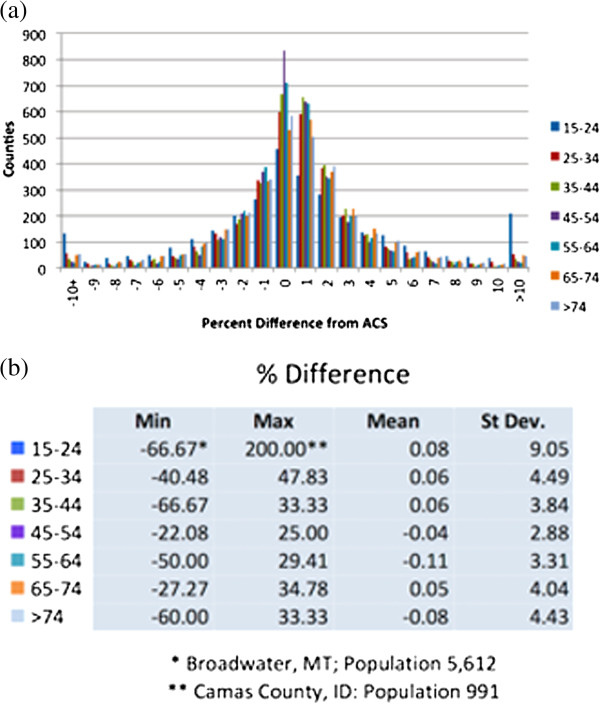
**County level agreement between synthetic population and the American Community Survey (ACS). (a)** Number of US counties with each percent difference in age of the head of household. **(b)** Mean and standard deviation over all counties of percentage differences by age of the head of household.

**Figure 2:**
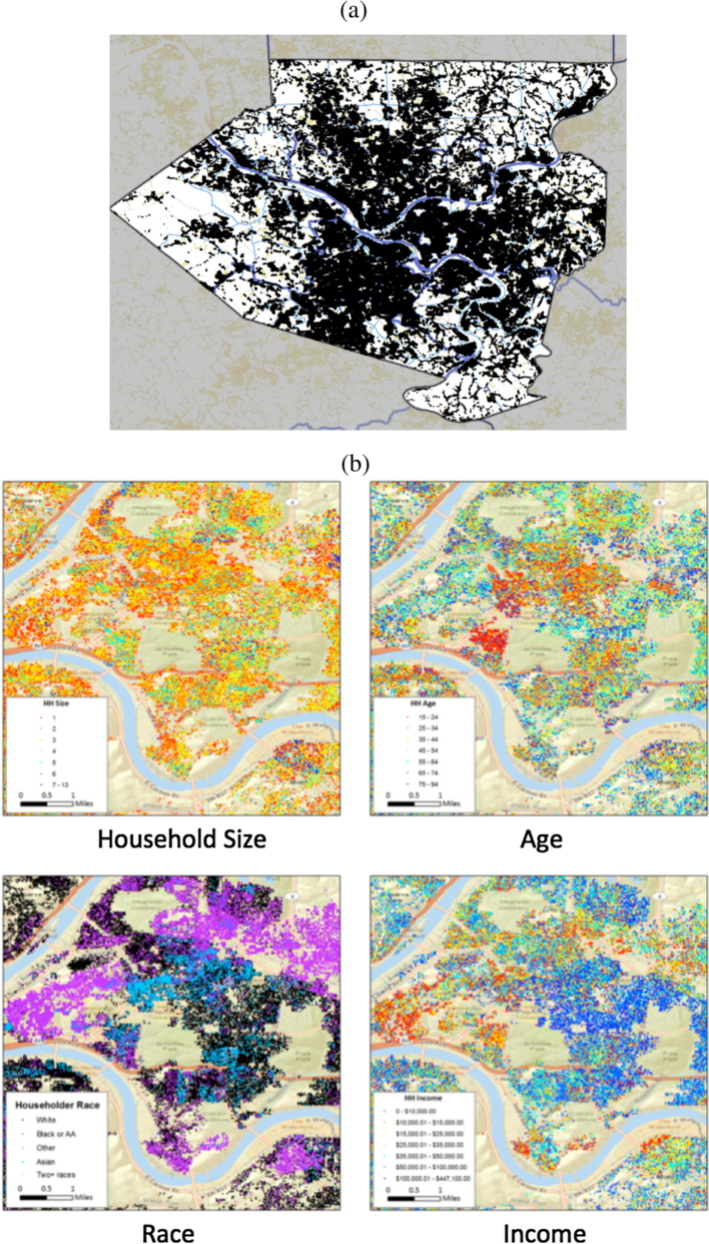
**Demographic features in Allegheny County synthetic population. (a)** Overall population density in Allegheny County. **(b)** Spatial distribution by household size, age of householder, race of householder, and household income.

The synthetic population closely matches the available census data for the United States with high spatial resolution. For example, the differences in the age of the head of household by county are shown in Figure 1. Overall the synthetic population differs from the ACS by less than 1% on this measure. Further detailed comparisons are provided in [31]. To illustrate the level of detail available for every county in the United States, Figure 2 shows various demographic distributions in the synthetic population for Allegheny County, PA.

The 2005–2009 U.S. Synthetic Population (Version 2) database is freely available, and synthetic populations are currently available from RTI for every state and county in the United States. Selected international locations, including Taiwan and Thailand, are available upon request from the authors. Users may apply FRED to other populations not included in the synthetic population database by using the file formats specified in [32].

### Discrete-time simulation

FRED performs a discrete-time simulation with time steps of one day, for any number of time steps. On each simulated day, each agent potentially interacts with the other agents who share the same activity locations. For example, school-age children in FRED interact with the same set of classmates at the school during each school day. If an infected agent interacts with a susceptible agent, there is a possibility of transmitting a disease from the infected agent to the susceptible agent. Each infection transmission event is recorded, making it possible to evaluate the effectiveness of several possible control measures and the impact on specific sub-populations. Agents may dynamically alter their daily activities, for example, by traveling or by deciding to stay home when sick.

The fixed simulation step of 1 day permits certain performance optimizations regarding scheduling the daily activities of agents and parallelizing the transmission of infection within places attended by disjoint sets of agents. The daily step size does not appear to be a severe limitation for running simulations that encompass several years for diseases with long latency periods, since the computation time per day depends primarily on the number of actively infectious individual on a given day. However, the daily step size may be a limitation for diseases with extremely short latency and infectious periods, or the simulation of short period (e.g. hourly) interventions.

### Agent model

Each agent maintains a record of its demographic information (e.g., sex, race, date-of-birth, current age, employment or school status, family income), health information (e.g., current health status, list of infections, date of infection, level of symptoms, infectivity, susceptibility, immunity status, at-risk status), locations for social activity (household, neighborhood, and school or workplaces as appropriate), and health-related behaviors (e.g., probability of getting a vaccine or staying home when sick).

By default, the demographic features of agents in FRED remain constant during a given simulation run. However, some research questions may address epidemic dynamics over many years (e.g., how will a pandemic affect the population immunity over the next several years). To address these questions, FRED includes as an option some limited forms of dynamic agent demographics including aging, births and deaths. Age-specific maternity and mortality rate can be specified in external files. If dynamic demographics is enabled, then an agent’s age may affect its activity pattern (e.g., school or work status) as well as the agent’s health status (e.g., eligibility for a vaccine). Children that achieve school age are assigned to schools and adults reaching working age are assigned to workplaces based on the attendance patterns in the agent’s neighborhood. Newborns are assigned to the same household as their mother. If an agent dies, it is removed from the population. Further refinements to the dynamic demographics model, including household recombination and migration patterns, are under development.

**Figure 3:**
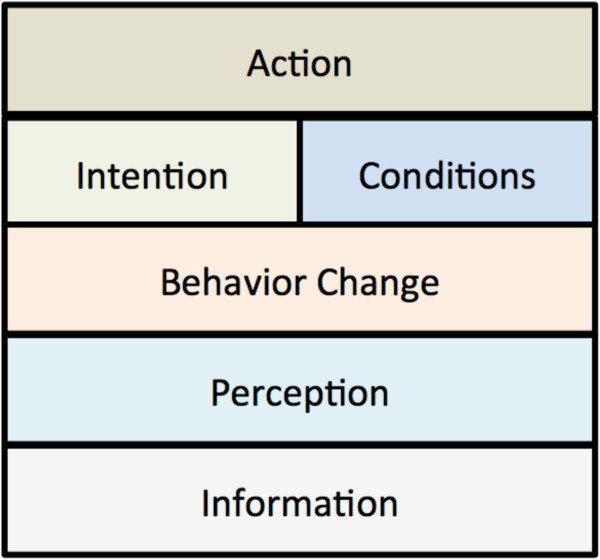
**Mechanisms for agent-specific health decision-making in FRED.** Agents can query the information layer to assess, for example, the current incidence, resulting in a perception (“how susceptible am I to the disease?”). Perceptions can be used by a behavior change model that determines whether to change the agent’s intention to perform the health-related behavior. These features permits the FRED developer to investigate a wide variety of alternative health behavior change models, including the Health Belief Model [36,37].

Agents in FRED may exhibit a number of optional health-related behaviors including staying home from work or keeping a child home when sick, and accepting a vaccine for oneself or for a dependent child. At each time step, the action taken by an agent involves an interaction between the intention of the agent to perform the behavior and one or more external conditions such as the availability of a vaccine. In addition to describing an agent’s intention as a simple probability as in previous models, FRED includes optional additional mechanisms for agent decision-making (Figure 3). As one example of using this flexible decision-making framework, FRED includes an implementation of the Health Belief Model, in which health behavior decisions are based on several specific constructs including perceived susceptibility, severity, benefits, and barriers [36]. These constructs are implemented as FRED perceptions and are combined into an agent-specific decision rule as described in [37]. The behavioral features of FRED are under active development, but some initial results showing the importance of behavioral heterogeneities within the population are available [27], described in the Results section below.

### Place model

FRED assumes that all disease-specific interactions among agents occur in a specific place, and each type of place represents a distinct environment for the spread of infection. The FRED framework includes a generic *Place* class that can be instantiated into subclasses as needed by the model developer for a particular study. The default types of places in FRED include *households*, *neighborhoods*, *schools*, and *workplace*s, but these are not intended as an exhaustive list of places that may be important sites of infection. *Neighborhoods* are defined on a grid with 1 km square cells. Agents tend to spend their neighborhood activities within their home neighborhoods, defined as the cell in which the agent’s household is located, but agents may also visit other neighborhood during a given day, according to a modified gravity model. FRED also provides optional *classrooms* (small mixing groups within a given school) and *offices* (small mixing groups within a given workplace). Non-workplace contacts in workplaces (e.g., customers) are not currently supported, but will be included in a future version of the framework.

Depending on an assigned *activity profile* (for example, *student*, *worker*, *retiree*, etc.), each agent maintains a default *daily schedule* of places that the agent visits on a regular basis (e.g., the agent’s household, neighborhood, school or workplace). Agent may visit different places depending on the day of the week, the time of year, or ad hoc travel plans. As in previous models [5-12], schools are closed on weekends and during scheduled summer holidays. Similarly, most workers do not visit their workplaces on weekends. However, some workers are designated as weekend workers, and they continue to visit workplaces on weekends. To reflect weekend schedules of schools and workplaces, the number of neighborhood contacts is increased by 50% on weekends [12].

### Disease model

The FRED framework supports the circulation in the population of one or more infectious diseases. Each disease is specified by a set of natural history, contact and transmission parameters (Table 2). The default distribution includes disease parameters for pandemic influenza derived from previously published influenza models [12]. All parameters are in Table 2 are user-modifiable based on the specific disease being modeled.

**Table 2:** User-modifiable disease-specific parameters

**Parameter type**	**Parameter**	**Definition**
**Natural history parameters**	Days latent	Discrete cdf for number of days between becoming exposed and becoming infectious
	Symptomatic rate	The probability of an infected person becoming symptomatic
	Days asymptomatic	Discrete cdf for number of days the agent is infectious but asymptomatic
	Days symptomatic	Discrete cdf for number of days the agent is infectious and symptomatic
	Immunity loss rate	Rate at which a person loses immunity after recovering from infection
	Mortality rate	The probability of an infected person dying
**Contact parameters**	Probability of staying home	The baseline probability that an agent stays home if the agent experiences a symptomatic infection.
	Household contact rates	The expected number of potentially infective daily contacts between an infectious agent and a susceptible agent in a household. All contact rates are positive real numbers.
	Neighborhood contact rates	The expected number of potentially infective daily contacts between an infectious agent and a susceptible agent in a neighborhood
	School contact rates	The expected number of potentially infective daily contacts between an infectious agent and a susceptible agent in a school
	Workplace contact rates	The expected number of potentially infective daily contacts between an infectious agent and a susceptible agent in a workplace.
**Transmission parameters**	Transmissibility	The transmissibility of disease relative to an arbitrary baseline set by calibration
	Asymptomatic infectivity	Multiplier for how infective an asymptomatic infected agent is, relative to an symptomatic agent
	Household transmission probability	A table of probabilities that a potentially infective contact between an infectious agent and a symptomatic agent in the same household actually results in an infection, given the age of the potential infector/infectee pair
	Neighborhood transmission probability	A table of probabilities that a potentially infective contact between an infectious agent and a symptomatic agent occurring in a neighborhood actually results in an infection, given the age of the potential infector/infectee pair
	School transmission probability	A table of probabilities that a potentially infective contact between an infectious agent and a symptomatic agent occurring in a school actually results in an infection, given the age of the potential infector/infectee pair
	Workplace transmission probability	A table of probabilities that a potentially infective contact between an infectious agent and a symptomatic agent occurring in a workplace actually results in an infection, given the age of the potential infector/infectee pair

FRED includes natural history and transmission parameters for pandemic influenza as used in previous models [5-12]. Contact parameters were calibrated for the FRED synthetic population using the methods described in [12]. For more details about these and other user-settable parameters, please see the FRED User Guide.

For a given agent, an infection is assumed to follow a user-specified temporal pattern, with the agent typically assuming the standard S-E-I-R pattern of *susceptible*, *exposed* (infected but not infectious to others), *infectious*, and *recovered* (or *removed*) states. It is also possible to specify the rate at which agents lose immunity after recovery. The distribution of the number of days spent in each state is specified by user-settable parameters. Thus disease patterns such as S-E-I-R-S or S-E-I-S can be modeled. FRED supports multiple strains circulating in the same population. The intensity and temporal trajectory of cross-immunity among strains, as well as its dependence on the genetic or antigenic distance between the strains, can be specified by the user. A detailed, equation-based intra-agent infection model is also available, so that the susceptibility, infectivity and symptoms of an agent can depend on the details of an agent’s exposure and treatment history. Other options, such as extending the *Intrahost* class to support more complex disease models, are described in the system documentation.

**Figure 4:**
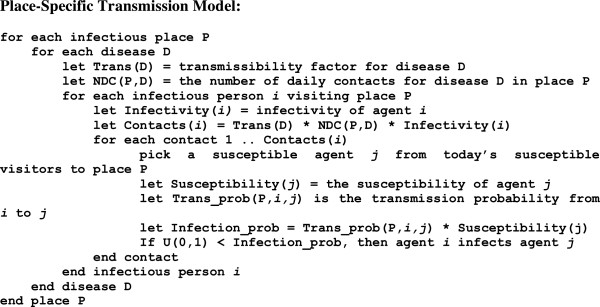
Pseudo-code for the place-specific transmission model in FRED.

If an agent is infectious on a given day, then any place the agent visits during that day is considered a *potentially infectious location*. The place-specific transmission model is described in Figure 4. Susceptible agents can only become infected at a potentially infectious location, so interactions among agents at non-infectious locations need not be simulated.

The rate of effective contacts (that is, the number of transmission events per infectious individual) in a given place depends on two place-specific parameters: the expected number of contacts per infectious person per day, and the probability that a contact transmits an infection. The expected number of contacts per day depends on the place type but not on the place size. For example, students in a small school are assumed to have the same number of contacts per day as students in a large school. The place-specific transmission probabilities depend on the ages of the agents involved. This permits the model to reflect, for example, that an infectious child in school is more likely to infect another student than to infect a teacher, even if the infectious child contacts both. By default these parameters are set as in previous models [5-12]. If an infectious agent has multiple contacts with a given susceptible agent (for example, as members of the same household or same classroom), each such contact is considered as an independent opportunity to transmit the infection.

Epidemics in FRED are initiated by seeding the population with one or more infections. These infections may either be assigned to members of the population selected randomly, or restriction to sub-groups defined by an age distribution or a selected geographical area. To account for the impact of an epidemic in populations external to modeled population, the user may specify a time-varying schedule of cases that causes FRED to seed new infections into the study area throughout the course of the epidemic.

### Performance and scalability

Computational efficiency is an important concern when modeling the potential interactions of millions of individuals. As in other large-scale epidemic models, FRED obtains much of its efficiency by focusing its transmission kernel only on the active set of infectious individuals and their interactions with susceptible individuals. FRED adopts a few additional significant optimizations:

1. Since every disease transmission occurs within a given place in FRED, we only apply the place-specific transmission model (Figure 4) to *potentially infectious* locations, that is, locations that are visited by at least one infectious individual during the current simulation day.

2. Once all potentially infectious locations are identified, the transmission model can be applied to all such locations of a given type in parallel. Simulating transmission in parallel in all infectious locations of a given type (e.g. all schools) ensures that no agent occurs in two such locations at the same time (e.g. all children attend at most one school). This avoids potential timing issues that may arise if all infectious locations were simulated in parallel.

3. FRED uses a shared memory multi-threaded parallel model implemented with OpenMP, which allows simulations on a quad-core computer with hyperthreading (i.e., many current laptops) to run approximately four times faster than with a purely serial implementation.

FRED requires between 750 and 1000 megabytes of memory per million simulated individuals. The exact amount of memory required depends on the demographic and geographic characteristics of the synthetic population, as well as the severity of the simulated epidemic. Simulations of an influenza spread like the H1N1 pandemic in a population of 1 million people takes less than two minutes on a typical dual-core laptop computer but the runtime will vary depending on the number of individuals infected during the epidemic and depending on which optional features are selected. On the supercomputer Blacklight at the Pittsburgh Supercomputer Center (an SGI Altix UV shared-memory architecture with up to 16 TB of shared memory), a simulated pandemic over the entire U.S. population requires approximately 200GB of memory and takes approximately 4 hours using 16 threads.

**Figure 5:**
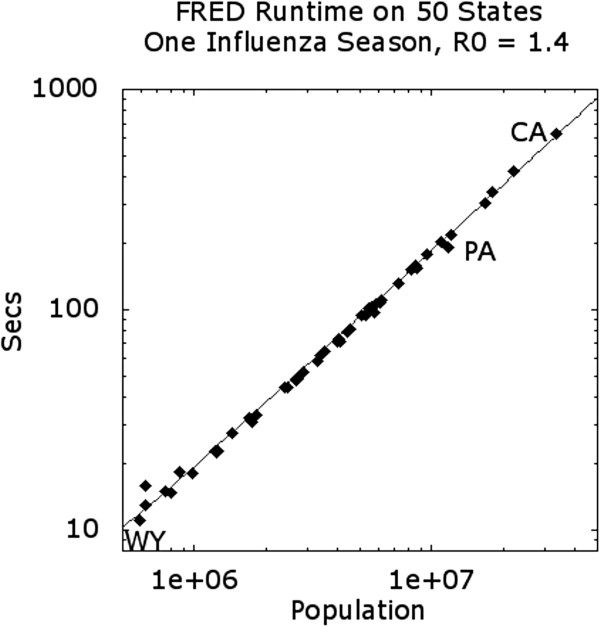
**Runtime in seconds as a function of population size (in millions of agents), in log-log scale.** Runtime is based on simulation of one influenza season in each of the 50 states and the District of Columbia. The states marked are WY (pop. approx. 500 K), PA (pop. approx. 11.8 M) and CA (pop. approx. 33.6 M). Observed runtimes were approximately 32.4 seconds per million individuals over the entire range of population sizes tested. Runs were performed using 16 threads on a 12-core Mac Pro with 64 GB of RAM, running at 2.93 GHz.

The synthetic populations for individual states and the District of Columbia range in size from approximately 600,000 individuals for Washington, DC to over 30,000,000 individuals in California. To demonstrate how FRED scales with population size, we performed FRED simulations of the 50 states and the District of Columbia using default influenza parameters and no intervention. Figure 5 shows that runtime scales linearly over two orders of magnitude in population size. Given FRED’s memory requirement of about 750 MB to 1GB per million agents, 30 million agents (e.g., California) can be simulated on a workstation with about 24 GB of memory.

### Implementation details

FRED is written in the C++ programming language and is released under the BSD 3-Clause Open Source License (http://opensource.org/licenses/BSD-3-Clause). The current distribution, available in Additional file 1, includes the FRED source code, documentation with installation instructions, tutorials on using the software, and detailed descriptions of all the configuration parameters. The documentation also describes the programming model and includes source level documentation and other details for developers who may be interested in extending the FRED framework for their own use.

The primary output file contains one line for each simulation day of the run, displaying a large selection of output variables. Optional additional files record the infection history of each infected individual, including the identity of the infecting individual, the place of infection and other details. The documentation describes the formats of the output files and how to modify the source code to include other variables if desired. FRED includes plotting scripts that display time series for any selected output variables.

FRED is distributed with sample synthetic populations, including Allegheny County (Pittsburgh), PA. Synthetic populations for other regions are available online as part of the 2005–2009 U.S. Synthetic Population (Version 2) [31]. Version 2.2.1 of FRED was used to produce the results in this paper. The latest version of the FRED distribution is available online at fred.publichealth.pitt.edu.

## Results

### Effects of schools closures during an influenza pandemic

**Figure 6:**
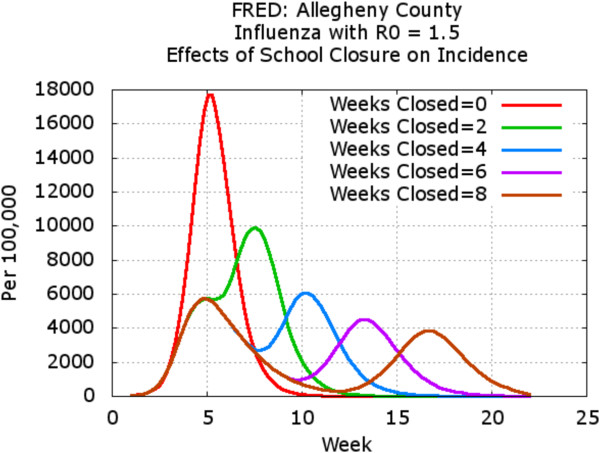
**Daily incidence curves for FRED pandemic influenza model under five school closure scenarios.** The baseline scenario assumed no school closures. For the other scenarios, individual schools in Allegheny County are closed the next day after 10 symptomatic students attended the school. The duration of the closure varied from 2 to 8 weeks. Regardless of the duration of the school closure, a secondary epidemic peak occurs when all the schools reopen.

**Figure 7:**
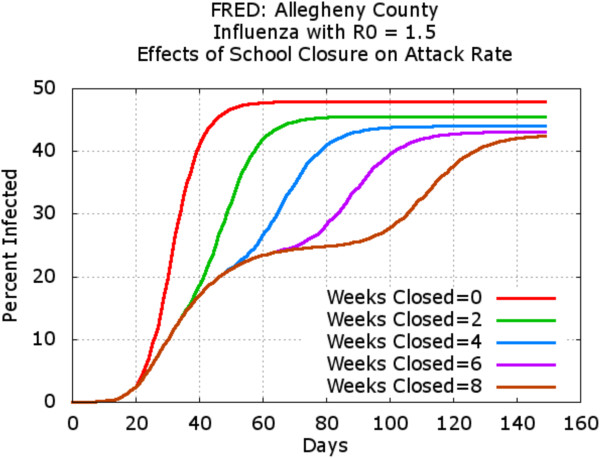
**Infection attack rates for five school closure scenarios.** The attack rate is significantly lower during the period corresponding to school closures, but the final attack rate is similar for all scenarios, reflecting the resurgence of the epidemic once schools reopen, as in [6].

To compare the current version of FRED with our previously published models, we reproduced studies from [6] that evaluated the potential effectiveness of alternative school closure policies during a pandemic influenza in Allegheny County, Pennsylvania. In particular, we considered policies that closed a given school when a number of sick students were observed at that school. The FRED software distribution includes a parameterization for pandemic influenza as used in previous models [5-12]. As described in detail in [12], place-specific contact parameters were calibrated using a 30–70 rule [3] in which 30% of all transmissions are assumed to occur in the household, 33% in the general community and 37% in schools and workplaces, and the fraction of transmissions that occur in schools is twice of those that occur in workplaces. The system was calibrated to reproduce a pandemic with a 50% Attack Rate (AR) in a completely susceptible population while satisfying the 30–70 rule. The baseline model assumed that 50% of sick individuals withdraw to their home and do not interact with anyone outside of the household, consistent with previous models [5-12],

In this study, a given school was closed when the number of sick children at that school reached a trigger value (fixed at 10 for this example). We compared the effects of varying the duration of the school closure once it was initiated at each school. Specifically, the duration of school closure was varied from 2 to 8 weeks, and once schools reopened, they did not close again. Figure 6 shows the daily incidence (number of new infections) for each scenario. Note that the curves for all school closure scenarios are essentially identical through the first 5 weeks of the epidemic, at which time the scenarios with shorter school closure durations begin to reopen schools. It can be observed that for all school closure policies, the epidemic temporarily abated when schools closed, but peaked again once the schools had reopened. The resulting attack rate (i.e. the total percentage of people that became infected through the course of the epidemic) is shown in Figure 7. While the daily incidence temporarily declined during the period corresponding to school closures, the final attack rate was similar for all scenarios, reflecting the resurgence of the epidemic once schools reopen. These results were consistent with our previously published model [6], although some details differed due to changes in the synthetic population model.

### Simulation of epidemics on all US counties

**Figure 8:**
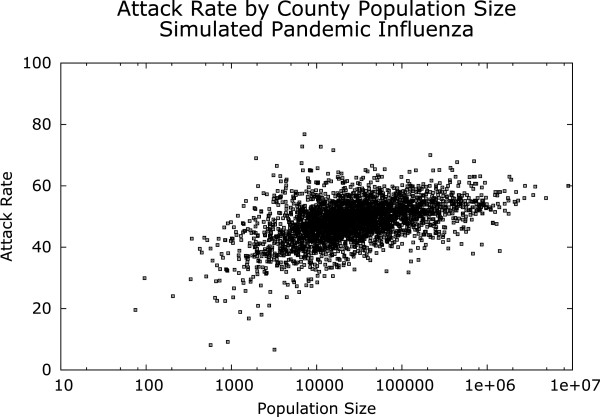
**Infection attack rate for 3142 counties in the United States, using FRED’s baseline pandemic influenza transmission parameters.** The plot shows the mean attack rate for each county over 20 stochastic simulations. The attack rate displays significant heterogeneity across US counties.

To illustrate the effect of regional differences on epidemic dynamics, we downloaded the synthetic population files for every county in the US and performed a FRED simulation of a baseline pandemic influenza epidemic in each county using the default parameters as discussed above. By using the same transmission parameters for all counties, we can see some of the effects of local heterogeneities in the population mixing patterns across the US. As expected, we observed a range of resulting attack rates for the 3142 counties tested (Figure 8). The mean attack rate (using 20 independent runs per county) for all counties was 48.4% (std. dev. 6.7). Counties with extremely small populations exhibited the extreme values for attack rate, with a minimum attack rate of 6.6% (for Catron County, NM; pop. 3202) and a maximum attack rate of 76.8% (Wade Hampton Census Area, Alaska; pop. 7203), perhaps showing the particularly strong effects of contact patterns in small populations. However, even among counties with populations over 500,000 a wide range of attack rates were observed, from a minimum of 37.9% for San Francisco County, CA (pop. 682,007) to a maximum attack rate of 68.0% for Hidalgo County, TX (pop. 701,751). These results suggest that health officials may want to consider the likely effect of interventions in the context of the local population structure.

**Figure 9:**
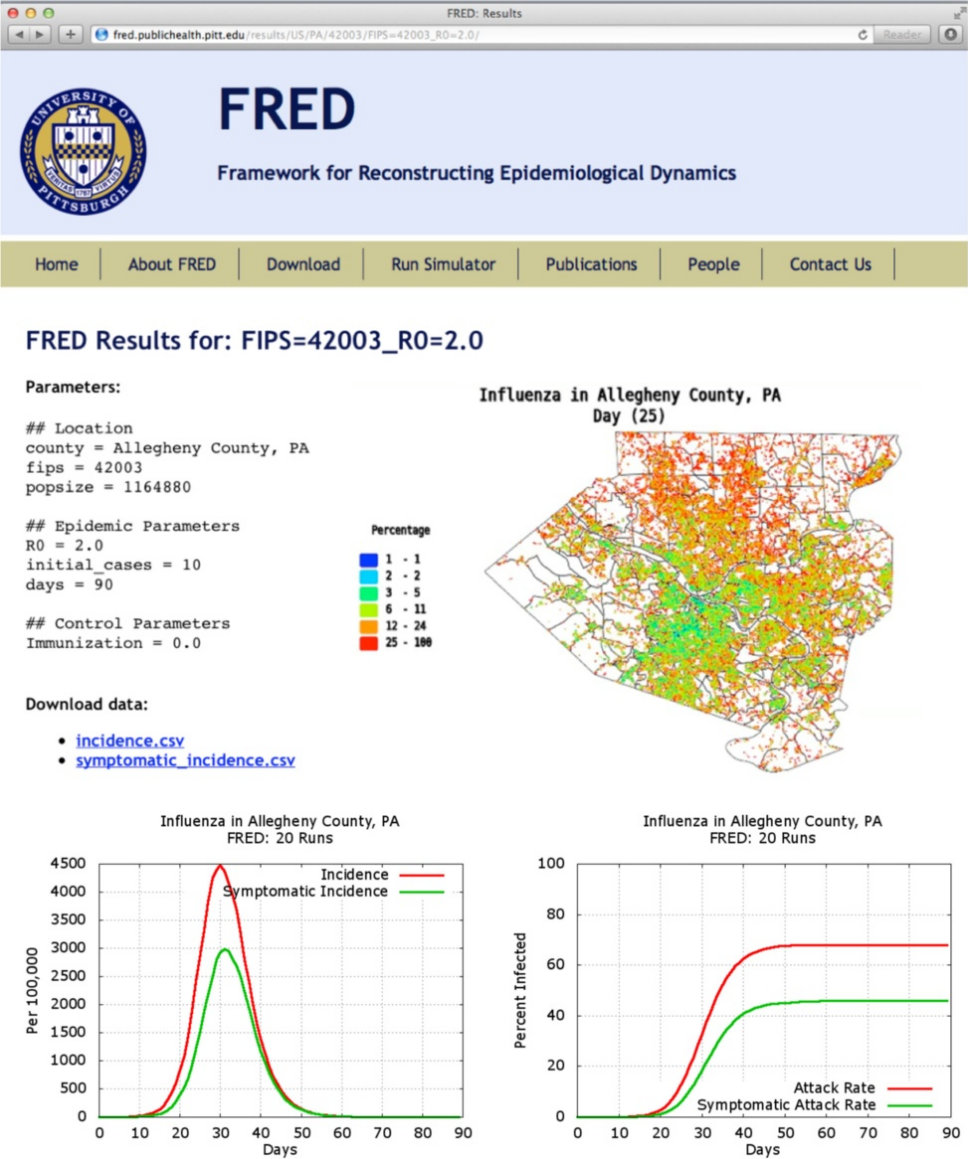
**Website showing results of FRED simulations.** Results available for an influenza simulation in Allegheny County, PA at fred.publichealth.pitt.edu. Similar results are available for every county in the US. Users can also specify epidemic parameters and control parameters for additional simulations, and can download data files to perform additional analyses.

To encourage further exploration of these simulation results, we have made FRED simulations of US counties available at fred.publichealth.pitt.edu. A user can browse thousands of previously run simulations (Figure 9), or run a new FRED influenza simulation with other combinations of epidemic parameters and control measures. In addition to incidence, prevalence and attack rate curves, the user can visualize results via maps and movies showing the epidemic dynamics for the given location via the GAIA webservice [Additional file 2]. Web users can also download FRED output data and perform their own analysis.

### Health-related behaviors and policies

A recent article [27] showed the possibilities for using FRED to investigate the impact of employment-related health policies on the health of both the workers directly involved and on the general population during an influenza pandemic. In this study, agents in FRED were assigned a probability of staying home based on the available of paid sick days (PSD) at their workplace. Based on data from the US Bureau of Labor Statistics [38], the model assumed that employees had access to PSD depending on the size of their workplace and that 72% of employees who had access to PSD and 52% of those without PSD stayed home when ill with influenza. The average amount of days off from work was also set on the basis of an observational study [39]. Simulations for Allegheny County, PA, showed that a large proportion (72%) of the workplace attack rate was due to exposure to other employees engaging in *presenteeism*, defined as going to work when symptomatic. It was also shown that providing universal PSD to all employees reduced workplace infections by 5.86%, with larger reductions in infection occurring in small workplaces (with 2–49 employees) than in large workplaces (with 500 employees or more). Finally, the results showed that providing one or two additional “flu days” (allowing employees with influenza to stay home) reduced workplace infections by about 25% and 39%, respectively. This study illustrates the importance of considering heterogeneities in the health-related behavior of individuals and in the workplace environments when considering the population-level impact of alternative policy interventions.

## Discussion

FRED simulations suggest that differences in population structure, spatial distribution, and other local factors can produce significant differences in the spread on infection disease among the counties in the U.S. We believe that these examples illustrate some of the advantages inherent in agent-based models that use data-driven population models, compared to simpler compartmental models that neglect heterogeneities present in real populations.

Several other epidemic simulation programs have been made available in open source form, including FluTE [40], EpiFire [41], GEM [42] and GSAM [43]. We believe that FRED offers an attractive combination of features that make it a valuable additional to the research community, including:

• A free, open source license

• Use of realistic, open-access, census-based synthetic populations

• Scalability from laptops to supercomputers

• Highly efficient simulation with populations up to hundreds of millions of agents

• An interface with the GAIA visualization system (http://gaia.psc.edu)

• Support for multiple circulating strains within the population, making it suitable for the investigation of virus evolution, for example, antigenic drift or the evolution of resistant strains.

FRED is under active development, and several additional features are planned for future versions that will extend its value as a tool for public health planning, including:

• Additional health-related behavior models, supporting the further study of how human behavior impacts potential public health control measures, and how health-related behaviors change over time.

• Long-term dynamic demographics of the agent population, such as migration patterns and household changes such as marriages and divorce, enabling the study of long-term health behavior patterns as well as chronic diseases such as tuberculosis.

• Vector-borne diseases, including dengue and malaria.

• A more flexible simulation time step.

• Automated workflows for advanced probabilistic sensitivity analysis [44].

In addition to the FRED web site, we have created an auxiliary tool called FRED Navigator [45] that allows the user to explore the effects of changing simulation parameters by interactively browsing through a database of simulation results. FRED Navigator is aimed at making FRED a practical tool for the public health user and a teaching tool for students in public health. We invite interested parties to contribute to the development of FRED and to extend its use as a tool for public health decision-making, research and education.

As with any model, users should take appropriate cautions to understand the limitations of FRED. Limitations of FRED include stochastic effects that limit the accuracy of the synthetic population especially in regions with very small populations [32], possible artifacts due to the selected time step resolution (one day), simplifying assumptions about travel patterns (gravity model), and the fact that estimates of contact rates and transmission probabilities are necessarily imperfect, even if based on estimates from the literature. Models created with FRED are stochastic, so results may vary from run to run, and some events, especially early in an epidemic, may depend on random choices such as the identity of the initial cases. Understanding the scope of variability in complex models such as FRED is an active area of research [44-46]. FRED supports the process of uncertainty analysis by providing workflow management scripts for setting up parameter sweeps and performing local sensitivity analysis.

## Conclusions

FRED (a Framework for Reconstructing Epidemic Dynamics) is a freely available open source epidemic modeling platform based on several previously developed influenza models. FRED simulates epidemics within a census-based synthetic population that reflects the specific population characteristics of a given region, including spatial distribution, race, age, and household income, along with realistic household, school, and workplace contact networks. FRED allows the flexible specifications of disease characteristics, intervention strategies, and a variety of health-related behaviors. These features make FRED a valuable tool for public health planners to explore possible epidemic scenarios in a specific jurisdiction and to evaluate the possible effects of interventions such as vaccination programs and school closure policies. We hope that the availability of FRED will contribute to the further development of modeling tools for public health decision support. We particularly welcome suggestions from the user community on ways to make FRED a more useful tool for planning public health responses to epidemics.

## Availability and requirements

**Project name:** FRED

**Project home page:**http://fred.publichealth.pitt.edu

**Operating system(s):** OS X, Linux, Windows (under Cygwin)

**Programming language:** C++, Python and Perl.

**Other requirements:** Optional plotting features require Gnuplot.

**License:** BSD Open Source License

**Any restrictions to use by non-academics:** None.

## Abbreviations

ACS: American community survey; FRED: A framework for reconstructing epidemic dynamics; PUMS: US census bureau’s public use microdata.

## Competing interests

The authors declare that they have no competing interests.

## Authors’ contributions

JJG, STB and RR designed the software architecture, based on original code developed by PCC and STB. WDW and PCC provided the interface to the synthetic population. JDP, NTBS, AF, DDG, AS, and TA contributed to the development of the software. HG contributed to the analysis of FRED components. DSB provided design goals and coordination for the project. JJG drafted the manuscript. All authors read and approved the final manuscript.

## Pre-publication history

The pre-publication history for this paper can be accessed here:

http://www.biomedcentral.com/1471-2458/13/940/prepub

## Supplementary Material

Additional file 1 **FRED Distribution Version 2.2.1.** This file contains the FRED distribution, including source files, installation instructions, required input files, and documentation. Unpack the file and see the file FRED/README.txt for further installation instructions. Click here for file

Additional file 2 **Quicktime movie.** GAIA Visualization of FRED simulation of Allegheny County. This file contains a Quicktime movie showing the prevalence of influenza in Allegheny County resulting from a FRED simulation calibrated to an R0 of 2.0. The movie was produced by the GAIA visualization tool (http://gaia.psc.edu). The FRED distribution contains scripts that automate the generation of GAIA visualization from FRED simulations. Click here for file
